# Genetic structure and local adaptation of *Nitraria sphaerocarpa* populations from arid northwestern China

**DOI:** 10.3389/fpls.2025.1623235

**Published:** 2025-09-03

**Authors:** Wenhui Ma, Jian Zhang, Haowen Tian, Yan Li, Hongxiang Zhang

**Affiliations:** ^1^ College of Ecology and Environment, Xinjiang University, Urumqi, China; ^2^ State Key Laboratory of Desert and Oasis Ecology, Key Laboratory of Ecological Safety and Sustainable Development in Arid Lands, Xinjiang Institute of Ecology and Geography, Chinese Academy of Sciences, Urumqi, China; ^3^ Xinjiang Key Laboratory of Conservation and Utilization of Gene Resources, Urumqi, China; ^4^ Specimen Museum of Xinjiang Institute of Ecology and Geography, Chinese Academy of Sciences, Urumqi, China; ^5^ College of Life Sciences, Xinjiang Agricultural University, Urumqi, China

**Keywords:** *N. sphaerocarpa*, ddRAD-seq, fragmented distributions, population genetics, environmental association analysis, environmental adaptive loci

## Abstract

**Introduction:**

Understanding the genetic basis of local adaptation in non-model species is one of the fundamental goals in ecological and evolutionary biology. Researches on the genetic mechanisms of local adaptation in desert plants is crucial to comprehend how species adapt to heterogeneous environments in arid regions under the background of climate warming.

**Methods:**

In this study, the typical superxerophytic constructive species of *Nitraria sphaerocarpa*, which is fragmentarily distributed in arid northwestern China, was sampled with 20 populations. A total of 10,828 high-quality SNPs were obtained by ddRAD-seq from 200 individuals of *N. sphaerocarpa* populations across the northwestern China, based on which the population genetic and local adaptation of *N. sphaerocarpa* was investigated.

**Results:**

The results showed that the population genetic diversity of *N. sphaerocarpa* was low. Twenty populations could be clustered into four lineages, which began to diverge in the Pleistocene. Mantel test showed that population differentiation was caused by geographical and environmental factors. Through gradient forest (GF), redundancy analysis (RDA) and niche comparison analyses, it was found that both temperature and precipitation factors affected the genetic differentiation of *N. sphaerocarpa* populations. Twenty-two loci associated with local adaptation were identified by environmental association analysis (EAA) using LFMM and RDA. Three successfully annotated environmental adaptive loci (EAL) were related to physiological processes in response to abiotic stresses such as drought, heat and cold.

**Discussion:**

In conclusion, the spatial genetic structure of *N. sphaerocarpa* populations showed a fragmented pattern in the latitude gradient. The main pressure of environmental adaptation was the changes of temperature and precipitation. Physiological adaptation appears to be an important mechanism in response to environmental stress.

## 1 Introduction

The fragmented distribution of desert plants is a key feature of the biogeographic patterns in arid northwestern China. This fragmented pattern was reported in *Nitraria sphaerocarpa* Maxim. and *N. roborowskii* Kom (Su et al., 2016), *Malus sieversii* (Ledeb.) M. Roem. (Zhang et al., 2021) and *Convolvulus gortschakovii* Schrenk (Jia and Zhang, 2021). Available hypotheses suggest that the species fragmented distribution are mainly caused by the changes of historical environment (Guu et al., 2010; Su et al., 2011; Li et al., 2012; Luqman et al., 2023) and environmental heterogeneity (Sang et al., 2022; Fu et al., 2023; Yan et al., 2023). Arid lands had experienced long-term of intensified aridification due to the retreat of the proto-Paratethys and global cooling (Bougeois et al., 2018), during which extensive deserts and mountains promote fragmentation of xeromorphic plants in arid northwestern China (Zhang et al., 2020). Meanwhile, the glacial-interglacial cycles occurred in the Pleistocene also strongly influences the geographical distribution of species, leading to local extinction, species migration and allopatric speciation (Hewitt, 2004; Willis and Niklas, 2004). For example, the geographical distribution and genetic structure of desert plants have changed significantly under the influence of Pleistocene climate changes (Ma et al., 2012; Su et al., 2012; Su and Zhang, 2013). In addition, orogeny also has greatly influenced on habitat fragmentation of species, such as Tianshan Mountains, Altai Mountains and Pamir Mountains in response to uplift of Qinghai-Tibet Plateau (Yang et al., 2011b). The uplift of mountains formed geological isolation with high mountains and deep valleys, which hinders the gene flow among species, leads to speciation, and further aggravates the fragmentation of distribution (Liu et al., 2006).

Historical climatic changes and geological events have shaped the early distribution patterns of species, laying the evolutionary foundation for the distribution of drought-adapted taxa. Against this backdrop, the present-day distribution and genetic structure of species are increasingly driven by contemporary climate change and local environmental factors. Current climate change is one of the most important driving factors of species distribution (Araújo and Rahbek, 2006; Hu et al., 2021). Under the heterogeneity of climate environment, plant populations are adapted to the local environment in order to survive (Kawecki and Ebert, 2004; Savolainen et al., 2013; Rellstab et al., 2015). Plant species adapted to local environments face selection pressures, and different selection pressures may lead to genetic variation and differentiation of species throughout the genome (Li et al., 2018). Local adaptation is common across plant populations (Leimu and Fischer, 2008), and is particularly evident in drylands or other environments with strong selective pressures (Baughman et al., 2019). For example, the distribution of fragmentation leaded to the genetic variation of *M. sieversii* (Zhang and Zheng, 2020), and the main environmental factor that determines the genetic variation was temperature (Zhang et al., 2022). Previous understanding of local adaptation or genetic structure differentiation mainly comes from a model plant *Arabidopsis thaliana* (Fournier-Level et al., 2011), but there is a lack of evidence on the genetic basis of local adaptation for non-model species. In the context of biodiversity research, the integration of non-model organism studies with the established genetic tools and methodologies of model organisms offers significant potential for large-scale data generation. Through advanced population genomics and landscape genomics analysis, the discovery of new population adaptive evolutionary molecular genetic mechanisms in non-model species is expected to be achieved in a broader and deeper dimension.

In recent years, with the development of high-throughput technology, the investigation of the genetic basis of local adaptation in non-model species has become the focus (Di Pierro et al., 2017). High-throughput sequencing technologies produce substantial high-density single nucleotide polymorphism (SNP) information, which provides opportunities for the development of population genetics research. Among them, Restriction site-associated DNA sequencing (RAD-seq) has become the most widely used genomic method for high-throughput SNP discovery in ecological and evolutionary studies of non-model organisms (Andrews et al., 2016). It has proven to be an effective and low-cost sequencing technology for obtaining genome-wide information, which is widely used because it is not constrained by “reference genome” (Miller et al., 2007; Wang et al., 2014; Zhou et al., 2018). RAD-seq can deeply explore the genetic lineage structure that the traditional molecular markers and a small number of DNA fragments cannot clarify (Emerson et al., 2010; Catchen et al., 2011; Lozier, 2014; Jeffries et al., 2016; Bai et al., 2018). More importantly, by comparing with annotated reference genomes, the genetic basis of species response and adaptation to environmental changes can be further revealed (Jia et al., 2020; Zhang et al., 2022).

The development of landscape genomics provides critical insights into the local adaptation of species which help us deepen our understandings of the response of natural populations to climate change (Savolainen et al., 2013). It integrates geographical and environmental information and uses a large number of genetic loci to quantify the degree of environmental heterogeneity of genetic variation in natural populations (Joost et al., 2007; Li et al., 2017; McKinney et al., 2017; Cushman et al., 2018). Identifying genetic variations associated with local adaptation is critical to elucidate the molecular mechanisms of adaptive evolution (Ahrens et al., 2019; Li et al., 2020). Through environmental association analysis (EAA), it can identify environmental and genetic factor driving local adaptation (Rellstab et al., 2015; Hoban et al., 2016). EAA uses high-throughput sequencing and high-resolution environmental data to correlate abiotic data with genomic data to explore the genetic basis of local adaptation of non-model species (Manel and Holderegger, 2013; Ellegren, 2014). Therefore, under multiple spatial scales and multiple geographical backgrounds of a single species, understanding how the environment shapes genetic variation can reveal the evolutionary molecular mechanism of local adaptation in natural populations (Sork et al., 2013; Gugger et al., 2021).

Drylands are defined as regions where precipitation is counter-balanced by land surface evapotranspiration. It is an important part of terrestrial ecosystem and covers about 40% of the terrestrial surface (Wang et al., 2022b). Drylands are one of the most vulnerable ecological areas characterized by low precipitation and high evapotranspiration (Reynolds et al., 2007), which leads to sparse vegetation, but hosts a variety of drought-adapted species (Maestre et al., 2021). Under the climate warming, the global water cycle is gradually accelerating (Wang et al., 2022a), which raises the frequency and severity of drought. The arid areas in northwestern China includes the Xinjiang, Hexi Corridor, and western Helan Mountain (Alxa Desert) (Dang et al., 2002) which is dominated by mountains and deserts, with dry climate and scarce precipitation. The plants inhabiting in this area have special structures and functions, such as *Haloxylon* and *Calligonum*, which provide important ecological benefits in windbreak and sand fixation, hydrological regulation, soil conservation and biodiversity protection (Cheng et al., 2020). Therefore, it is of great significance to study the adaptation mechanism of plants in this ecosystem. Especially in the context of global warming, the adaptive strategies of desert plants can provide valuable guidance for future wild resource protection and stress resistance breeding.

**Figure 1:**
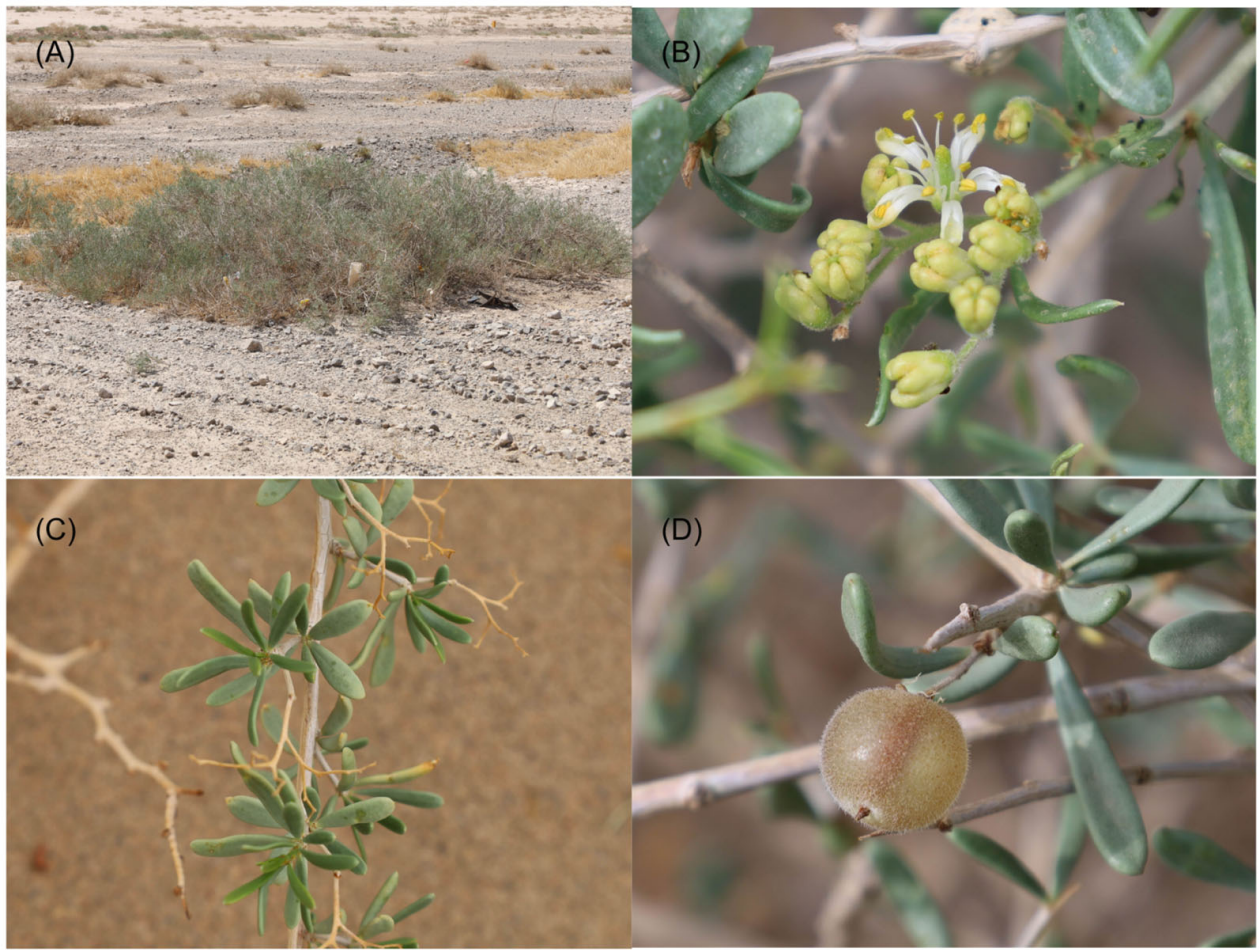
Morphology of *Nitraria sphaerocarpa.* **(A)** Habitat; **(B)** Flowers; **(C)** Leaves; and **(D)** Fruit.

**Figure 2:**
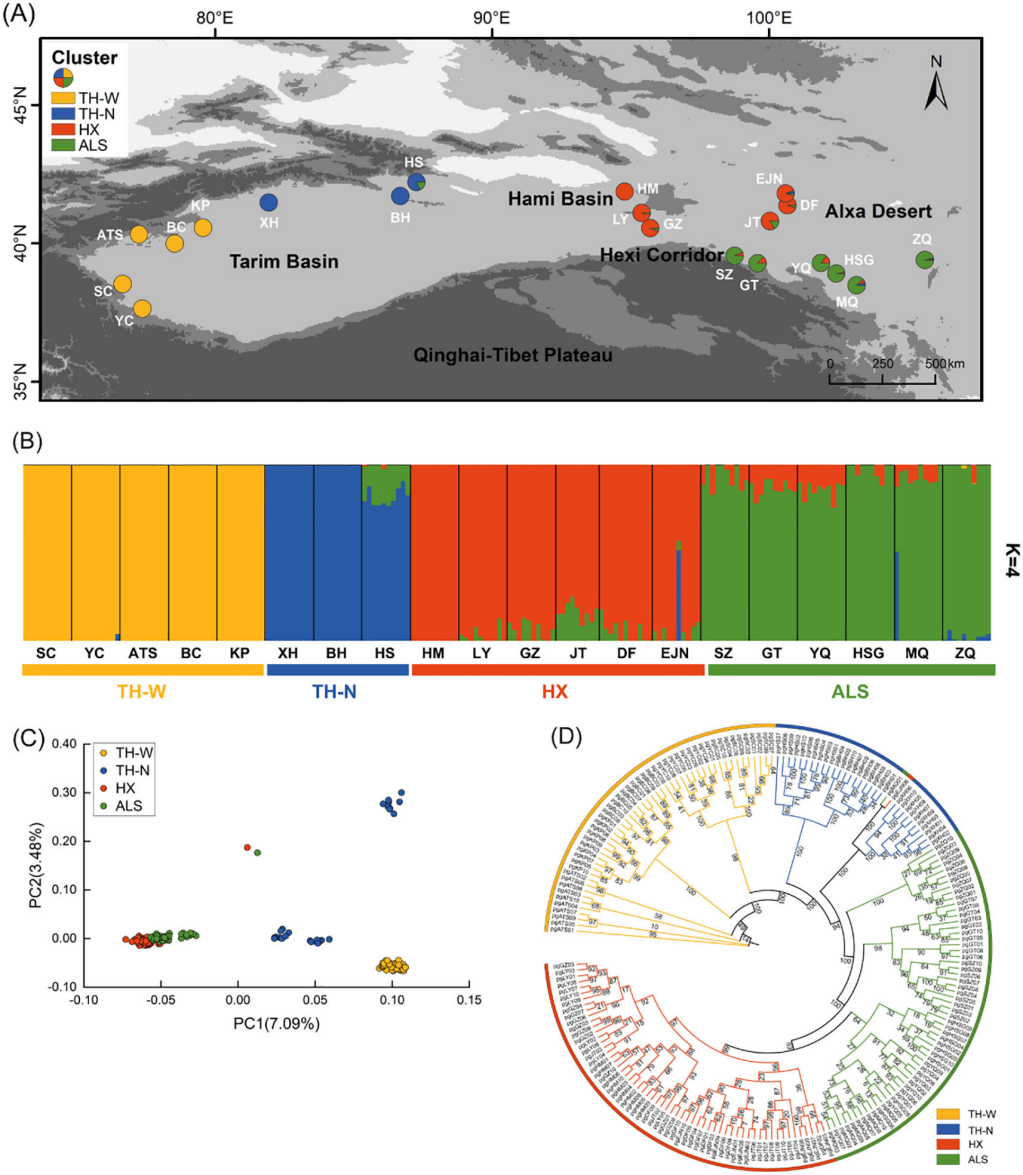
Population genetic structure of *N. sphaerocarpa*. **(A)** Geographic distribution of 20 populations (pie chart) where colors represent ancestral components inferred by ADMIXTURE (K = 4). **(B)** Population genetic structure estimated by ADMIXTURE analysis with K = 4. Each vertical bar represents an individual, the x-axis represents each population and four clusters and the y-axis represents the proportion of ancestors contained in each individual. **(C)** PCA. Each point represents an individual in which colors distinguish groups. **(D)** Phylogenetic tree of *N. sphaerocarpa* based on IQ-tree employing the maximum likelihood (ML) method. Degree of confidence was evaluated by ultrafast bootstrap approximation with 1,000 replicates. A color represents a cluster and the number on the node represents the degree of confidence.

In the arid regions of northwestern China, *N. sphaerocarpa* exhibits a fragmented distribution pattern and strong adaptability, making it an ideal model for studying genetic differentiation and local adaptation of plants in arid environments. More specifically, *N. sphaerocarpa* is a xerophytic shrub, which is common in gravelly desert, piedmont, and gravelly sand substrates (**Figure 1A**). It is one of the important constructive species in arid northwestern China, mainly distributed in Xinjiang, Gansu, and Inner Mongolia, and plays an important role in maintaining the stability of the ecological environment (Su and Zhang, 2013). For example, *N. shaerocarpa*, with its near-ground clumped growth form, helps reduce soil erosion. Its well-developed root system stabilizes sand and traps sediments, while long-term litter accumulation creates a “fertile island effect,” enhancing soil organic matter, supporting plant diversity, and maintaining ecological balance. At present, *N. sphaerocarpa* owns fragmented distributions extending from the west of the Tarim Basin, through the Hami Basin and the Hexi Corridor, to the Alxa Desert in the east, (**Figure 2A**), making it an ideal material for studying the adaptation mechanism of plants in extremely habitats. It has white flowers and leathery leaves (**Figures 1B, C**), and chiefly reproduces sexually by means of insect pollination (Wang et al., 2012). Compared with other *Nitraria* species occurred in China, *N. sphaerocarpa* has the unique morphological characteristic that the exocarp becomes dry membrane and expands to form micro-transparent ball when the fruit matures (**Figure 1D**). *Nitraria sphaerocarpa* has excellent characteristics such as salt tolerance, drought resistance, sand fixation and soil improvement (Li et al., 2004b). Thus, this shrub plays a vital role in windbreak and sand fixation, soil and water conservation, as well as in desert management.

Taken together, previous observations and the heterogeneous habitats (e.g., soil type, aridity) across the species’ distribution range suggest that *N. sphaerocarpa* populations may exhibit genetic differentiation and local adaptability. However, the genomic basis underlying such potential adaptations remains largely unexplored. We used population genomics and landscape genomics, based on ddRAD-seq and environmental association analysis (EAA), to analyze the response and adaptation mechanism of desert constructive plant *N. sphaerocarpa* to arid environment. We aimed to: (1) evaluate the genetic diversity and population structure related to fragmented distribution patterns; (2) clarify the relationship between environmental factors and genetic variation; and (3) analyze the molecular mechanism of population adaptive evolution of *N. sphaerocarpa*. This study helps provide a theoretical basis for elucidating the molecular genetic mechanism of local adaptive evolution of *N. sphaerocarpa* driven by environmental factors, and shed lights on the adaptive evolution of other plants in extreme desert environment.

## 2 Materials and methods

### 2.1 Sampling

The spatial distribution of *N. sphaerocarpa* was initially referenced from the Flora of China, the Chinese Virtual Herbarium (CVH; https://www.cvh.ac.cn/), and prior research (Su and Zhang, 2013). In the summer of 2023, fresh leaves of 200 *N. sphaerocarpa* samples were collected from 20 different sites, covering nearly its entire geographical range in China (**Table 1**, **Figure 2A**). No specific permissions were required for sampling and collection from these localities. Voucher specimens are deposited in the Herbarium of the Xinjiang Institute of Ecology and Geography Chinese Academy of Sciences (XJBI) (**Supplementary Table S1**). Plant identifications were conducted by Hongxiang Zhang and Haowen Tian. Among the sampled populations, eight (pgSC, pgATS, pgYC, pgBC, pgKP, pgXH, pgBH, and pgHS) were from the Tarim Basin, one (pgHM) was from the Hami Basin, seven (pgLY, pgGZ, pgJT, pgSZ, pgGT, pgHSG, and pgMQ) were from the Hexi Corridor, and four (pgDF, pgEJN, pgYQ, and pgZQ) were from the Alxa Desert (**Supplementary Table S1**). Within each population, 10 individuals were sampled at least 10 m apart from each other to avoid collecting from the same female parent. Fresh leaves were harvested and dehydrated using silica gel.

**Table 1:** Population locations and summary of genetic statistics of *N. sphaerocarpa*.

Cluster	Population	Longitude(°E)	Latitude(°N)	*Ho*	*He*	*π*	*F_IS_*
TH-W	pgSC	76.69	38.55	0.1333	0.1405	0.0278	0.0314
	pgATS	77.28	40.34	0.1506	0.1487	0.0297	-0.0378
	pgYC	77.40	37.66	0.1531	0.1517	0.0293	-0.0277
	pgBC	78.57	40.01	0.1370	0.1445	0.0274	0.0402
	pgKP	79.59	40.58	0.1520	0.1527	0.0288	-0.0155
TH-N	pgXH	81.96	41.49	0.1470	0.1412	0.0284	-0.0868
	pgBH	86.72	41.73	0.1415	0.1464	0.0270	0.0277
	pgHS	87.30	42.23	0.2052	0.1851	0.0307	-0.1218
HX	pgHM	94.83	41.89	0.2045	0.2108	0.0286	0.0245
	pgLY	95.44	41.12	0.2428	0.2253	0.0308	-0.0840
	pgGZ	95.75	40.57	0.2116	0.2135	0.0285	0.0087
	pgJT	100.08	40.83	0.2242	0.2210	0.0295	-0.0228
	pgDF	100.71	41.40	0.2281	0.2184	0.0298	-0.0573
	pgEJN	100.64	41.82	0.2069	0.2169	0.0276	0.0360
ALS	pgSZ	98.80	39.57	0.2341	0.2184	0.0311	-0.0797
	pgGT	99.63	39.30	0.2435	0.2225	0.0311	-0.1016
	pgHSG	102.47	38.93	0.2092	0.2109	0.0286	0.0032
	pgMQ	103.21	38.50	0.2110	0.2157	0.0273	0.0105
	pgYQ	101.92	39.31	0.2256	0.2191	0.0291	-0.0406
	pgZQ	105.68	39.42	0.1986	0.2006	0.0288	-0.0002
Mean				0.1930	0.1902	0.0290	-0.0247

*Ho、He、π* and *F_IS_* represent observed heterozygosity, expected heterozygosity, nucleotide diversity, inbreeding coefficient, respectively.

### 2.2 ddRAD-seq and SNP filtering

Molecular samples of *N. sphaerocarpa* were submitted to Paisano Biotechnology Co., Ltd (Shanghai, China) for double-digest restriction-site-associated DNA sequencing (ddRAD-seq). The total genomic DNA was extracted by the kit method (Tiangen DP305 Plant Genomic DNA Kit), and the quality of DNA was detected by 0.8% agarose gel electrophoresis, while DNA was quantified by ultraviolet spectrophotometer (Thermo Scientific NanoDrop 2000/2000c, Thermo Fisher Scientific). Whole-genome DNA was digested with a restriction enzymes, DpnII and MspI, and size-selected DNA fragments (~300–500 bp) were used for library construction, following standard ddRAD-seq protocols. After sample quality inspection, the standard Illumina TruSeq Nano DNA LT library preparation experimental process was utilized to construct a library with an insert fragment of 400 bp, followed by Paired-end (PE) sequencing based on the Illumina NovaSeq sequencing platform for Next-Generation Sequencing (NGS). Subsequently, post-sequencing, the original offline data (raw data) was filtered to generate high-quality sequences (high quality data) using fastp v0.20.0, employing a sliding window method with a window size of 5 bp and a step size of 1 bp. Reads with a joint read length ≤ 50 bp were excluded during this filtering process. The obtained high-quality data was aligned to the reference genome *N. sibirica* (Ma et al., 2023) using BWA-MEM (0.7.17-r1188) (Li and Durbin, 2009) mem with default parameters. Moreover, Picard v1.107 (Zhao and Assoc Comput, 2018) and GATK (McKenna et al., 2010; DePristo et al., 2011) were employed to enhance the precision of SNP prediction.

We used PLINK v1.90 (Purcell et al., 2007) with the parameters “indep-pairwise 50 10 0.2” to filter out a Linkage Disequilibrium–pruned (LD-pruned) SNP set with a minor allele frequency < 0.05, significant deviation from Hardy-Weinberg equilibrium (HWE, 0.0001 level), linkage loci (statistical correlation coefficient *R^2^* > 0.2 between gene loci) and SNP loci with individual deletion in the sample (plink –maf 0.05 –geno 0.2 –hwe 0.0001 –indep-pairwise 50 10 0.2). After filtration, 10,828 high-quality SNPs were yield for subsequent analysis.

### 2.3 Population genetic diversity, structure and differentiation

The observed and expected heterozygosity (*Ho* and *He*), nucleotide diversity (*π*) and inbreeding coefficient (*F_IS_*) were calculated using PLINK v1.90 (Purcell et al., 2007) with the filtered SNP dataset. The population differentiation (*F_ST_* statistic) was measured using the software Arlequin v3.5.2.2 among *N. sphaerocarpa* populations (Excoffier and Lischer, 2010). The ADMIXTURE analysis, principal components analysis (PCA) and phylogenetic inferences were employed to explore the population genetic structure. Firstly, the optimal genetic clusters of the *N. sphaerocarpa* populations was identified by the cross-test error value (CV) of assumed clustering value K (1 ≤ K ≤ 10) implemented in ADMIXTURE v1.3.0 (Alexander et al., 2009). Next, PCA was conducted in GCTA (Genome-wide Complex Trait Analysis) v1.26.0 (Yang et al., 2011a) to assess the genetic variance. Finally, IQ-TREE v2.0.3 (Nguyen et al., 2015) was utilized for constructing the maximum likelihood (ML) tree with GTR+F+G4 model. Additionally, 1,000 ultra-fast bootstrap tests were employed to determine the node support rate.

### 2.4 Demographic history

Filtered 10,828 SNPs were further screened at 10 kb intervals using VCFtools v0.1.16 (Danecek et al., 2011), resulting in 4,648 SNPs. Logistic regression was performed with the *DIYABC* v1.2.1 software and Approximate Bayesian Computation (ABC) (Cornuet et al., 2008) with a 95% confidence interval. One percent of the observed data that had the closest match to the simulated data was used to estimate posterior probabilities (PP) for each evolutionary event. The scenario with the highest PP was considered the optimal solution, representing the most probable ancestral source of the four geographic groups. To align with the ADMIXTURE outcomes and the geographic distribution of 20 *N. sphaerocarpa* populations, eight competition scenarios were designed. These scenarios included: (1) the simultaneous divergence of four lineages from a shared ancestor at time point t3 (scenario 1; **Supplementary Figure S2**); (2) the initial split of the common ancestor into two lineages at t3, with the emergence of a third lineage at time t2 on one branch and a fourth lineage at time t1 on the same branch (scenario 2-4; **Supplementary Figure S2**); and (3) the division of the common ancestor into two lineages at t3, with subsequent re-diversification into new lineages at t2 and t1 on separate branches, respectively (scenario 5-8; **Supplementary Figure S2**).

### 2.5 Isolation by distance and isolation by environment

Based on the availability of data, environmental data, consisting of 19 climatic variables for 1979-2013, bio 1-bio 19, were obtained from PaleoClim (http://www.paleoclim.org) at a resolution of 30s (about 1 km) (Brown et al., 2018). Climate data corresponding to the geographic coordinates of 20 *N. sphaerocarpa* populations were extracted using the R package “raster” (Hijmans and Etten, 2010). To mitigate the biased estimation and false significance level of the model coefficients caused by multicollinearity, Pearson correlation analysis was performed using SPSS (IBM Corp. Released 2020. IBM SPSS Statistics for Windows, Version 27.0. Armonk, NY: IBM Corp) among 19 variables. Variables with a correlation coefficient exceeding |r| > 0.9 were deemed highly correlated and thus removed to reduced autocorrelation (Dormann et al., 2013). Ultimately, 13 environmental variables were retained for further analyses (**Supplementary Table S3**).

To explore the impact of geographical and environmental factors in the formation of spatial genetic differentiation, mantel tests were conducted to examine isolation by distance (IBD) and isolation by environment (IBE) with significance determined using 999 permutations in the R package “vegan” (Dixon, 2003). Three distinct datasets were employed for the analysis: (1) genetic distances were estimated using the formula *F_ST_*/(1 – *F_ST_*) (Rousset, 1997); (2) the geographical distance between populations was calculated using the R package “geosphere” (Hijmans, 2024); and (3) environmental distance was quantified by calculating the Euclidean distance across all scaled environmental variables.

### 2.6 Environmental association analyses

We ran the non-parametric, machine learning regression tree approach known as gradient forest (GF) in the R package “gradientForest” (Ellis et al., 2012b) to evaluate the effects of environmental factors on population genetic variation. This approach enables the analysis of nonlinear associations between environment and allele variables by dividing the allele frequency data into “split values” according to environmental gradients (Breiman, 2001). The split importance metric quantifies the amount of variation explained, appearing high along the gradient where allelic frequency change is large. In the GF analysis of the relationship between genetic variation and environmental factors, the split importance values are accumulated to produce a ladder curve for allele frequency change along the environmental gradient (Ellis et al., 2012a). Consequently, GF analysis was performed on 13 environmental variables (**Supplementary Table S3**) and allele frequency data to evaluate the relative importance of each environmental variable through *R^2^* weighted importance, and to visualize the cumulative importance of alleles along environmental gradients.

### 2.7 Niche comparison analyses based on environmental variables

To quantitatively analyze the niche differentiation and environmental driving forces of *N. sphaerocarpa* populations distributed across four lineages, kernel density plots were first generated for each four lineages utilizing 13 environmental variables through the R package “ggplot2”. Subsequently, the key factor values of population distribution points were extracted for PCA to elucidate the primary factors influencing the niche differentiation of four lineage populations (Yan et al., 2021).

### 2.8 Environmental adaptive loci

The environmental association analysis (EAA) method was utilized to detect the genomic loci of *N. sphaerocarpa* populations that are adapted to the local environment. However, EAA has limitations, as they might lead to high rates of false positives (Frichot and François, 2015). To prevent false positives, one effective approach is to integrate multiple methods and utilize gene ontology (GO) analyses to connect positively identified SNPs with gene function (Rellstab et al., 2015).

Firstly, a univariate latent factor mixed model [LFMM; (Frichot et al., 2013)] was implemented in the R package LEA (Frichot and François, 2015) to find out environmental adaptive loci (EAL) linked with six environmental variables (**Supplementary Table S3**; bio 1, bio 2, bio 4, bio 11, bio 15 and bio 18). By determining the number of clusters through ADMIXTURE results, LFMM with latent factors (K = 4) was conducted to elucidate the population structure within the genotype data. For each environmental variable, we ran with 5 repetitions and burning of 5,000 steps followed by 10,000 iterations. The |z|-scores were averaged to enhance the genetic-environment association, with a false discovery rate (FDR) of 1% applied to identify significant loci (Frichot and François, 2015).

Secondly, redundancy analysis (RDA) was performed through the rdadapt function in the R package “vegan” (Dixon, 2003). A total of 10,828 SNP loci were considered as dependent variables, with six environmental factors (**Supplementary Table S3**) as explanatory variables. The first four axes (explaining 88% of the genetic variation) were used to assess genetic variation across SNP loci in the genome based on the degree of interpretation of different RDA axes for the variation. After that, the false positive probability (*q*) of FDR of SNP loci was calculated, ensuring a stringent threshold of *q* < 0.01 to identify candidate adaptive loci.

Finally, environmental adaptive loci (EAL) were identified using the combination of LFMM and RDA methods. The eggNOG (Huerta-Cepas et al., 2019) was used for GO annotation and enrichment to detect the gene function of the hypothetical EAL. Specifically, (1) the genetic coordinates of candidate SNPs were isolated by comparing the candidate loci with the reference genome of *N. sibirica* (Ma et al., 2023); (2) the corresponding protein sequences were obtained by searching the above genetic coordinates in the reference genome; and (3) the genome GO annotation was performed by submitting all the extracted protein sequences to eggNOG.

## 3 Results

### 3.1 ddRNA-seq and SNP calling

We constructed a ddRAD-Seq library for each sample, resulting in a total of 200 libraries and sequenced 150-bp paired-end from 200 individual *N. sphaerocarpa*, generating a total of 9.92 GB sequencing data from these individuals. This produced an average of 9,606,459 bp (range 6,464,386-19,313,540 bp) raw sequence reads per sample. Following the initial sequencing, the raw data underwent filtering to obtain high-quality sequence reads, yielding an average of 9,346,120 bp (6,114,880-19,102,036 bp). Subsequently, an average of 8,815,090 bp (5,822,354-18,132,655 bp) high-quality reads were successfully aligned to the reference genome. The identified SNPs were then subjected to filtration based on linkage disequilibrium (LD), resulting in the final 10,828 high-quality SNPs for further analysis.

### 3.2 Population structure and diversity

*Nitraria sphaerocarpa* among 20 populations exhibited low genetic diversity (*He=*0.1902). Notably, population pgGT, situated in ALS cluster, demonstrated the highest genetic diversity, as evidenced by the highest *Ho* (0.2435), *He* (0.2225) and *π* (0.0311). In contrast, TH-W cluster, pgSC, displayed the lowest level of genetic diversity (*Ho* = 0.1333 and *He* = 0.1405; **Table 1**). Overall, the populations in Tarim Basin exhibited low genetic diversity (e.g., pgSC, pgXH), while those in Hexi Corridor and Alxa Deser (e.g., pgJT, pgGT, pgYQ) showed higher genetic diversity, indicating a trend of increasing genetic diversity from west to east within the study area. Moreover, the genetic differentiation relationships among different populations were further explored through pairwise genetic differentiation (*F_ST_*). The *F_ST_* values calculated among the 20 populations ranged from 0.00 (pgGZ and pgLY) to 0.59 (pgBH and pgXH), with an average of 0.30 (**Supplementary Table S2**). Importantly, the TH-W lineage consistently displayed high pairwise *F_ST_* values in comparison with other genetic clusters, providing preliminary evidence of genetic differentiation and limited gene flow. Conversely, populations within the HX and ALS lineages exhibited relatively low *F_ST_* values, reflecting weak genetic differentiation and implying the presence of ongoing or historical gene flow between them.

The cross-validation error rate supported the optimal clustering of K = 4 for *N. sphaerocarpa* populations (**Supplementary Figure S1A**). These four genetic clusters were primarily correlated with the geographic distributions of the populations (**Figure 2A**, **Supplementary Table S1**). Specifically, a highly homogenous genetic cluster (TH-W) encompassed five populations from the western Tarim Basin, while three populations from the northern Tarim Basin were grouped into a distinct genetic cluster (TH-N) that exhibited genetically close to the western Tarim Basin. One Hami Basin, three western Hexi Corridor, and two western Alxa Desert populations formed a single cluster (HX), highlighting genetic similarities within these regions. Furthermore, the remaining six populations fell into one genetic clusters (ALS). Notably, a significant genetic divergence was observed between TH-W and the other genetic clusters. Further evidence indicates limited gene flow between these groups, whereas gene flow was clearly evident between the HX and ALS cluster (**Figures 2A, B**). Interestingly, when K = 3 (**Supplementary Figure S1B**), the populations from the Hexi Corridor and Alxa Desert merged into a single genetic group, further supporting the genetic relationships observed within the study populations. The population structure was further confirmed by the PCA and phylogenetic tree topology. PCA analysis revealed that only TH-W formed a clearly distinct cluster. TH-N showed internal subdivision into three subgroups but remained genetically differentiated from other populations. In contrast, HX and ALS populations displayed extensive admixture with highly overlapping distributions and scattered individuals, indicating substantial gene flow between these populations (**Figure 2C**). Additionally, the phylogenetic analysis using the IQ-tree method revealed that almost all individuals within each population clustered together, forming four well-supported evolutionary branches (**Figure 2D**).

### 3.3 Demographic history

**Figure 3:**
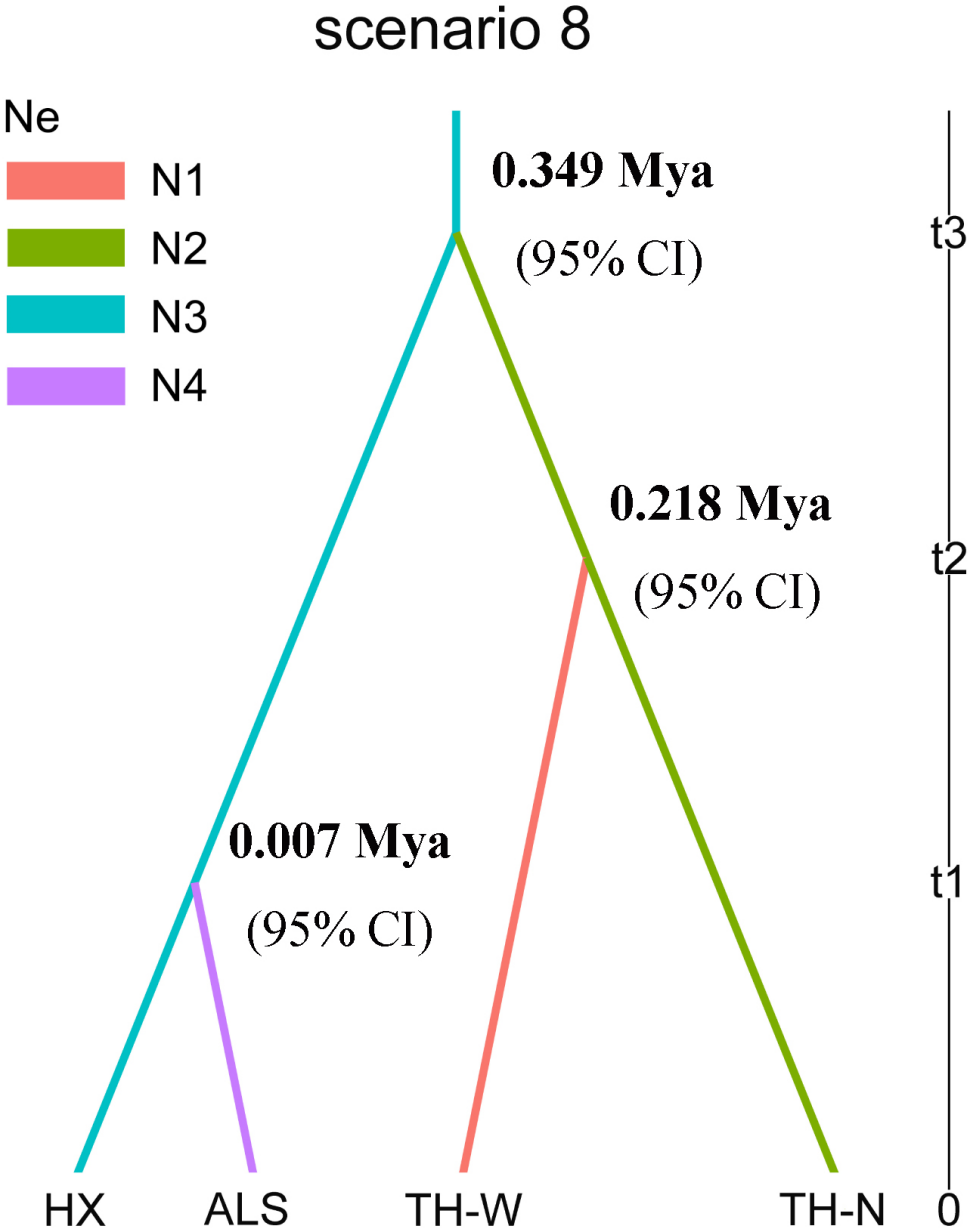
Scenario 8 of population history of four lineages in *N. sphaerocarpa* with *DIYABC*. N1, N2, N3 and N4 represent the effective population size of the four lineages. T1, t2 and t3, divergence times for the depicted event.

In the analysis conducted using *DIYABC* software, it was found that scenario 8 demonstrated the highest consistency with the historical differentiation among the four lineages under study (**Figure 3**, **Table 2**). The generation time for *N. sphaerocarpa* was determined to be three years (Su and Zhang, 2013). The divergence time between each lineage was estimated by multiplying the generation time of *N. sphaerocarpa* by the expected values of time parameters (t1, t2, and t3) obtained from the parameter estimates of scenario 8. Specifically, the TH-N and HX lineages diverged from a common ancestor approximately 0.349 (3×116407×10^-6^≈0.349) million years ago (Mya; 95% CI) during the middle Pleistocene. Following this, the TH-W lineage separated from the TH-N lineage around 0.218 (3×72631.7×10^-6^≈0.218) Mya (95% CI) in the late middle Pleistocene, while the ALS lineage differentiated from the HX lineage at 0.007 (3×2413.52×10^-6^≈0.007) Mya (95% CI) in the Holocene (**Figure 3**, **Table 3**). The effective population size (Ne) of the HX lineage (N3) was estimated to be 1.06×10^7^. Furthermore, the effective population sizes of the TH-W, TH-N and ALS lineage were estimated to be 1.71×10^5^, 3.68×10^5^ and 0.25×10^5^ for lineage N1, N2 and N4, respectively (**Table 3**).

**Table 2:** 8 scenarios of votes and the optimal model based on *DIYABC*.

Votes model 1	Votes model 2	Votes model 3	Votes model 4	Votes model 5	Votes model 6	Votes model 7	Votes model 8	Selected model	Post proba
14	24	3	32	4	52	66	305	8	0.667

**Table 3:** Parameter estimation (point estimates) of optimal scenario (scenario 8) based on *DIYABC*.

Parameter	Expectation	Median	Quantile_0.05	Quantile_0.95	Variance
N1	170932	169299	45490	297667	2841450000
N2	367704	375125	109624	570734	12058200000
N3	10601600	9893100	4337280	18996700	11896400000000
N4	25322.8	24631.7	6396	49776	103784000
t1	2413.52	2358.5	1191.99	3866.8	746004
t2	72631.7	70310.8	23585.5	123607	440270000
t3	116407	116248	47698.1	191206	1666450000

### 3.4 IBD and IBE

**Figure 4:**
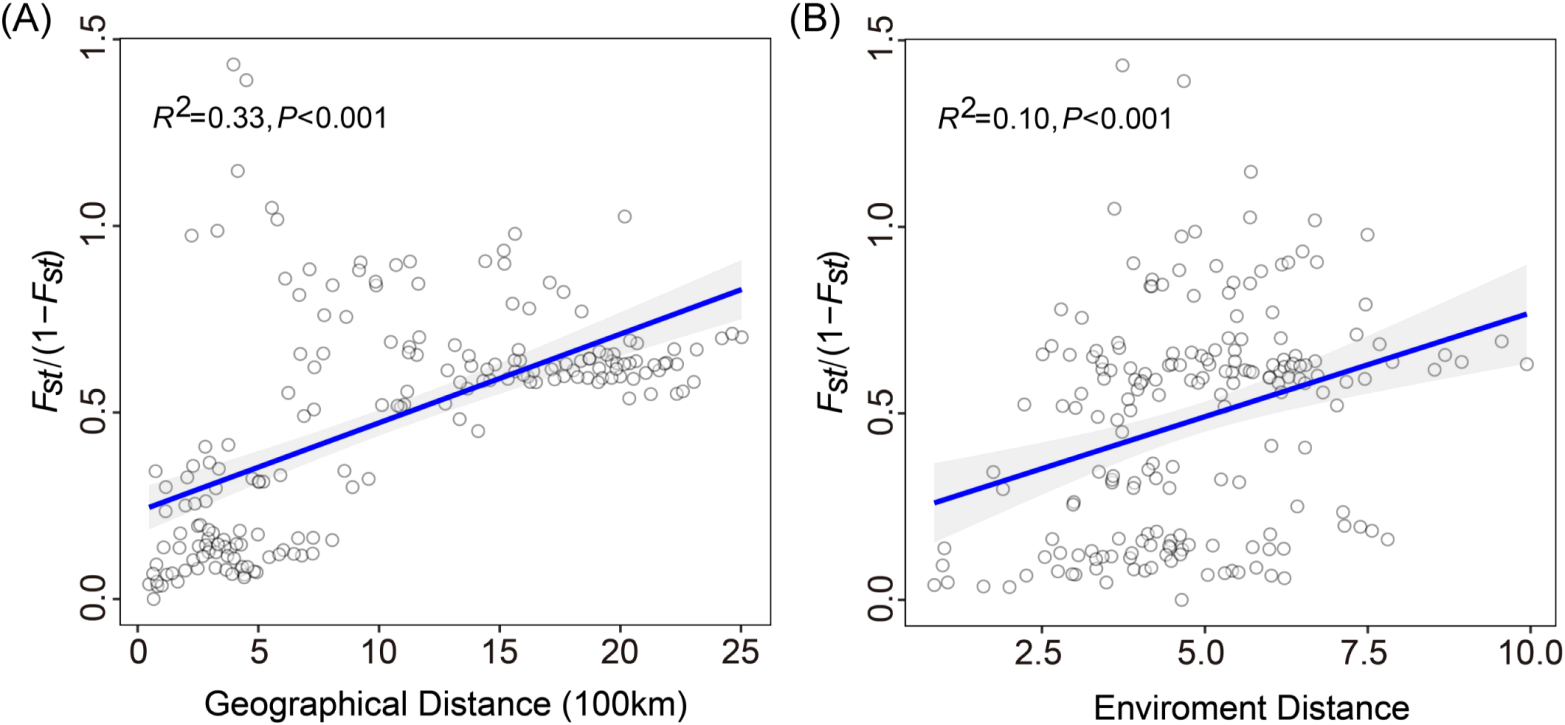
Mantel tests of genetic distance and geographical distance or environmental distance. **(A)** Genetic distance is significantly associated with geographic distance and **(B)** environmental distance.

Mantel test indicated a significant correlation between pairwise *F_ST_*/(1 - *F_ST_*) and geographic distance (*R^2^* = 0.33, *p* < 0.001; **Figure 4A**), supporting the presence of isolation by distance (IBD). This suggests a potential pattern of local adaptation among populations that are geographically distant. Furthermore, the positive correlation observed between genetic distance and environmental distance (*R^2^* = 0.10, *p* < 0.001; **Figure 4B**) among the 20 populations confirmed the pattern of isolation by environment (IBE). These findings provide evidence that genetic distance was strongly influenced by both geographical and environmental distances.

### 3.5 Genetic variation associated with environmental factors

**Figure 5:**
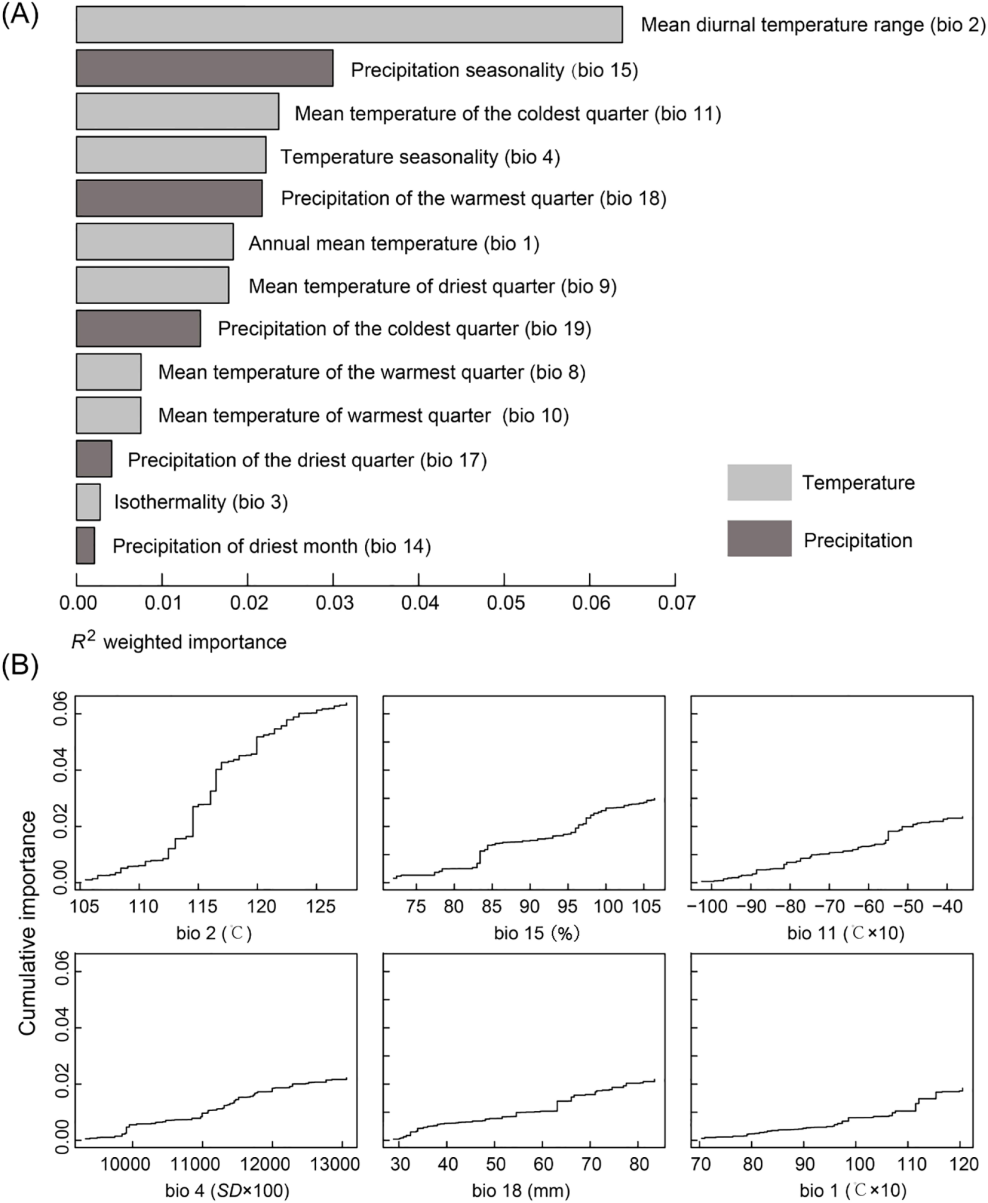
Gradient forest (GF) analysis. **(A)** *R^2^* weighted importance of environmental variables. **(B)** Cumulative importance of allelic change along the first six environmental gradients.

**Figure 6:**
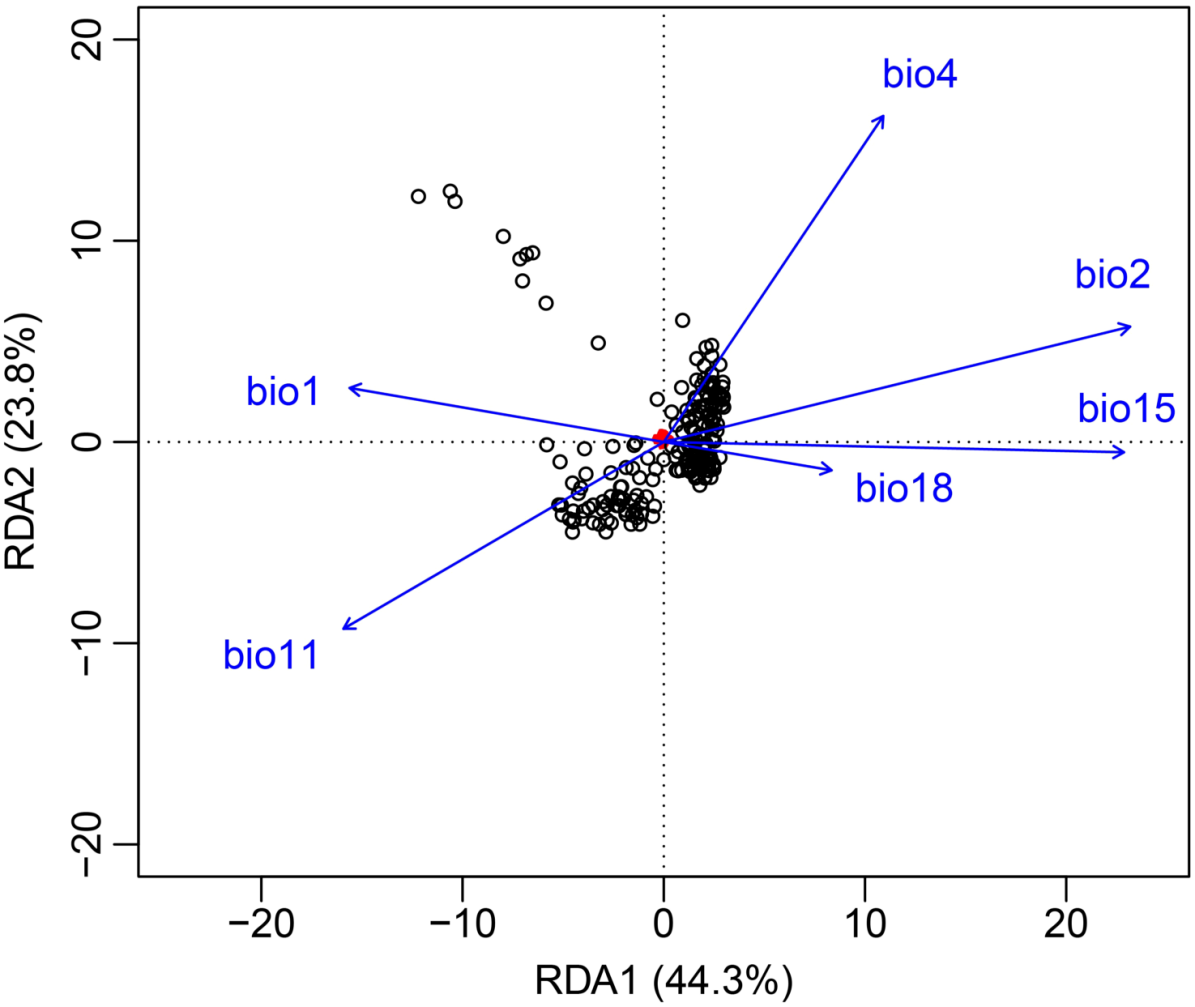
Redundancy analysis (RDA). It showed the relationship between environmental variables and genetic variations. Small red points are single nucleotide polymorphism (SNP). Individuals are black points.

The GF model revealed a significant correlation between genetic variation and the mean diurnal temperature range (bio 2), with its *R^2^* weighted importance value ranking highest, and other important factors included precipitation seasonality (bio 15), mean temperature of coldest quarter (bio 11), temperature seasonality (bio 4), precipitation of warmest quarter (bio 18) and annual mean temperature (bio 1) (**Figure 5A**). The remaining seven environmental factors exhibited low *R^2^* weighted importance values, suggesting that their impact on genetic variation was relatively minor. **Figure 5B** illustrated the cumulative importance of all allele frequency changes with the environmental gradient across the top six climate variables. Moreover, through RDA analysis, it was also found that there was a significant correlation between genetic variation among populations and the six environmental factors (*p* = 0.001). The joint contribution of axes 1 and 2 in the RDA accounted for 68.1% of the total genetic variation (RDA1: 44.3%, RDA2: 23.8%; **Figure 6**). The ranking of each environmental factor in terms of population genetic variation as revealed by RDA was roughly in agreement with the GF analysis.

### 3.6 Niche comparison analyses

**Figure 7:**
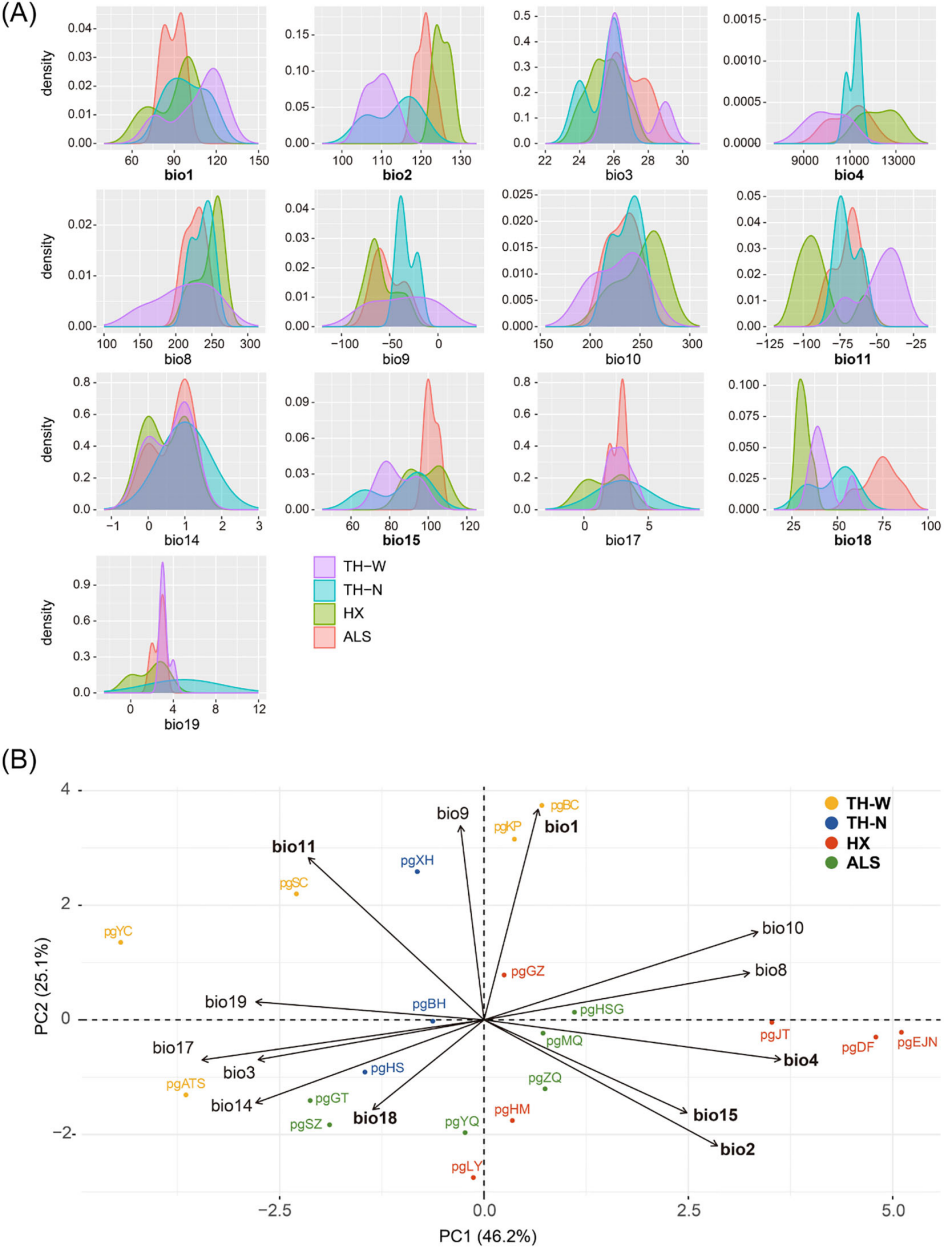
Niche comparison analyses. **(A)** Kernel density plots for 13 environmental parameters for *N. sphaerocarpa* showing niche differences among the four lineages. **(B)** PCA-env analysis. The bold variables indicate the 6 environmental variables selected for RDA analysis.

Kernel density plots were first generated to illustrate the frequency distributions of 13 environment variables (**Supplementary Table S3**) in four distinct lineages. The results indicated that the four lineages exhibited significant differences in both temperature- and precipitation-related environmental variables, with precipitation factors showing more pronounced differentiation. (**Figure 7A**). Meanwhile, PCA-env analysis revealed that both temperature and precipitation variables were associated with genetic polymorphism, with temperature exhibiting a stronger correlation. Specifically, temperature seasonality (bio 4) prominently influenced niche differentiation in the first component, while annual mean temperature (bio 1) played a key role in driving differentiation in the second component. In addition, axis 1 (PC1) and axis 2 (PC2), accounted for a substantial 71.3% of the overall variability, with PC1 explaining 46.2% and PC2 explaining 25.1% of the total variance (**Figure 7B**). These findings were further supported by the outcomes of the GF and RDA analyses, which consistently indicated that *N. sphaerocarpa* experienced environmental adaptive pressures primarily driven by fluctuations in temperature and precipitation, demonstrating concordance across multiple analytical approaches.

### 3.7 Identifying genetic loci associated with local adaptation

94 and 81 SNPs were found to be significantly associated with six environmental variables by LFMM and RDA analysis, respectively, of which 22 common SNPs identified between both methods were assumed as environmental adaptive loci (EAL) (**Supplementary Figure S3**). Functional annotation was carried out on 22 SNPs, indicating that 3 SNPs (loci LG03_1166504, LG04_5700855, and LG11_4056481) were matched to genes with known functions. Specifically, the locus LG03_1166504 matched to pentatricopeptide repeat-containing protein At5g13270, which is relates to the cold response of the plant (Li et al., 2023a); the locus LG04_5700855 was annotated as encoding protein phosphatase 2C (PP2C), which functions as a key regulator in various hormone signal transduction pathways, particularly those involving abscisic acid (ABA) signaling. PP2C members can respond to various biotic and abiotic stresses, and also regulate organ development and seed germination (Rodriguez, 1998; Chen et al., 2021); and the locus LG11_4056481 was associated with R3H-associated N-terminal domain, which is crucial for immune response, cell death and signal transduction in plants (**Table 4**).

**Table 4:** Functional annotation of the three candidate loci under local adaptation.

Loci ID	Environmental variables	Description	Evalue	Score
LG03_1166504	bio 2; bio 11	Pentatricopeptide repeat-containing protein At5g13270, chloroplastic	0.0	1018.0
LG04_5700855	bio 1; bio 2; bio 4; bio 11	phosphatase 2C	0.0	1320.0
LG11_4056481	bio 1	R3H-associated N-terminal domain	2.28e-128	370.0

## 4 Discussion

### 4.1 Effect of historical and current environment factors on fragmented distribution pattern of *N. sphaerocarpa*

The fragmented distributions could explain evolutionary mechanism of genetic divergence for *N. sphaerocarpa*. At present, *N. sphaerocarpa* is distributed with a fragmented pattern in arid northwestern China (**Figure 2**). The fragmentation of *N. sphaerocarpa* was resulted from the combined effects of historical and current environmental factors. The drying climate during the Quaternary was considered to be a driving factor for significant genetic differentiation among populations in *N. sphaerocarpa* (Su and Zhang, 2013). During the Quaternary, northwestern China experienced the long-term process of increasing aridification, especially in the Pleistocene (Guo et al., 1999). In this period, periodic cold and aridification were associated with glacial epoch (Xu et al., 2010). In the early Pleistocene, the climate began cold, and several aridification expansion events occurred in northwestern China (Guo et al., 1999). At 0.8-0.6 Mya, northwestern China entered into the largest glacial period, and the climate became colder and drier than ever before (Shi et al., 2005). The desert and gobi continued to expand, and the degree of drought further increased during the middle Pleistocene. In this study, the TH-N and HX lineages diverged from a common ancestor at 0.349 Mya (95% CI; middle Pleistocene) and the TH-W lineage diverged from the TH-N lineage at 0.218 Mya (95% CI; late middle Pleistocene) (**Figure 3**, **Table 3**). Thus, we speculate that climate change, especially the extreme cold and dry climate during glacial period, reduces the viability of *N. sphaerocarpa* and limits its distribution. The distribution range might have been reduced and fragmented, so that the intraspecific populations could be divided into several groups (Su and Zhang, 2013). The gradually reduced and isolated habitats of *N. sphaerocarpa* leaded to genetic differentiation among isolated regions. According to the population structure of *N. sphaerocarpa* in the existing distribution area in our study, 20 populations could be reasonably divided into four geographical groups (**Figure 2**). Because of the commonality of historical experiences in the same region, other desert plants may exhibit similar patterns of population structure fragmentation, such as *Nitraria roborowskii* Kom (Su et al., 2016), *Reaumuria soongarica* (Pall.) Maxim. (Li et al., 2012) and *Populus euphratica* Oliv (Wang et al., 2011).

Here, we found a significant pattern of isolation by distance (IBD) among the 20 populations of *N. sphaerocarpa*, providing a unique genetic structure for fragmented populations (**Figure 4A**). Studies have been shown that there was a significant correlation between genetic distance and geographical distance of *N. sphaerocarpa* (Su and Zhang, 2013). Under the fragmented distribution pattern and long-distance range, the gene flow between populations of *N. sphaerocarpa* was limited. Also, in views of the topography of northwest China, landscape barriers might be the reason for hindering gene flow. Major landscape barriers in the study region include the arid Tarim Basin and the Alxa Desert, along with surrounding mountain ranges such as the Tianshan and Altai Mountains, which together impose significant physical and ecological constraints on gene flow. In addition, the fragmented desert–steppe ecotone, exemplified by patchy habitats along the Hexi Corridor, further restricts the continuous distribution of populations. In addition, the different degrees of genetic differences might be related to the biological characteristics of the species. The seed set percentage of *N. sphaerocarpa* was 33.85%, which was lower than that of other species of *Nitraria* (Wang et al., 2012). Its mature fruits were relatively scarce, and in contrast to the fleshy berries of other *Nitraria* species, the seeds of this species were less prone to being collected and dispersed by animals (Li et al., 2013a). These factors will increase genetic differentiation between populations by limiting gene flow. Human activities (e.g., overgrazing, agricultural expansion, and infrastructure development) cannot be ignored as they have contributed to population fragmentation, reducing gene flow and increasing the risk of maladjustment of local species in response to environmental changes.

Also, a significant pattern of isolation by environmental (IBE) was shown among these 20 populations besides IBD (**Figure 4B**). The results of this study showed that the primary environmental adaptation pressures faced by *N. sphaerocarpa* was from fluctuations in temperature and precipitation conditions. Among the environmental variables, GF analysis identified that mean diurnal temperature range (bio 2) was the most important environmental factor affecting the genetic variation of *N. sphaerocarpa* populations (**Figure 5**), highlighting the significant role of temperature fluctuations in shaping the genetic structure. Generally, the area with large diurnal temperature range has sufficient daytime sunshine, high temperature and strong solar radiation, which is very beneficial to the photosynthesis of plants, while low temperature at night causes weak plant respiration, less energy consumption and more organic matter accumulation (Peng et al., 2013). In China, the diurnal temperature range shows a spatial differentiation pattern of more in the northwest and less in the southeast (Kong, 2020). In addition, precipitation was also an important driving factor for genetic variation and local adaptation of *N. sphaerocarpa* populations, such as precipitation seasonality (bio 15; **Figure 5**). Water deficit was a critical condition, which imposed a strong selective pressure promoting local adaptation in plants (Biehler and Fock, 1996; Eveno et al., 2008). For *Nitraria* plants, water conditions (precipitation and groundwater) are the main constraints on their growth (Li et al., 2004a). The arid area of northwest China is far away from the ocean, with dry climate and scarce precipitation. There are spatial and temporal differences in rainfall, mainly concentrated in summer. The average annual precipitation is about 156.36 mm. Among them, the average annual precipitation in the Tarim Basin in southern Xinjiang is only 74.2 mm (Chen et al., 2023). To adapt to the geographical changes of these ecological factors, the population of *N. sphaerocarpa* has evolved a matching adaptive genetic variation distribution pattern under the action of local adaptation. Finally, the adverse effects of climate change on desert plants will largely depend on the capacity of plants to tolerate temperature and precipitation changes. In sharp contrast, the niche differentiation of the *N. sphaerocarpa* appears to be more strongly driven by precipitation-related variables rather than temperature (**Figure 7**). This difference reveals the distinct dimensions of environmental adaptation in *N. sphaerocarpa*, as reflected in genetic variation and niche differentiation: the former captures historical evolutionary responses at the genomic level, while the latter represents contemporary distribution patterns constrained by climatic suitability. Collectively, these findings underscore the dual role of temperature and precipitation in shaping the adaptive of *N. sphaerocarpa*.

Taken together, there are two main modes to construct the fragmented distribution pattern of *N. sphaerocarpa* populations. The first mode involves the formation of fragmented distribution pattern. *N. sphaerocarpa* is widely distributed in the northwest arid area, with the drying climate during the Quaternary leading to habitat shrinkage or fragmentation. In addition, the remote distance of the distribution area and poor seed dispersal ability limit the gene flow between populations, which further aggravates the genetic differentiation between populations. The second mode focuses on the maintenance of the fragmented distribution pattern. Climate change poses challenges for organism growth, particularly for desert plants. Precipitation and temperature have been identified as being primary drivers of local adaptation and species distribution limitations, similar to findings in *Stipa breviflora* (Yan et al., 2023). so that *N. sphaerocarpa* still exhibits a fragmented spatial genetic pattern. Overall, historical climatic changes appear to have played a dominant role in the formation of the fragmented distribution pattern of *N. sphaerocarpa*, while contemporary climate variability may be more influential in its maintenance. Future research could further clarify the relative contributions of historical and modern climatic factors across different spatial scales by integrating high-resolution geographic, climatic, and paleoenvironmental data, combined with spatial modeling and experimental validation.

### 4.2 Genetic mechanism of *N. sphaerocarpa* populations adaptation to environment

Throughout the course of species evolution, plants have developed a set of mechanisms to adapt to heterogeneous environments. Firstly, leaf morphology is particularly responsive to environmental variation and has demonstrated substantial plasticity during the course of plant evolution. Thus, the morphological characteristics of leaves serve as a vital indicator of how plants have adapted to different environmental conditions (Gielwanowska et al., 2005; Kröber et al., 2015). For instance, in arid environments, various plant species exhibit distinct morphological adaptations. *N. tangutorum* reduced leaf length, leaf and epidermal cell thickness (Zhao et al., 2020), while *N. sibirica* adjusted by sinking and shrinking stomata and thickening the cuticle of leaves. Second, in terms of physiological adaptation mechanism, *N. tangutorum* and *Caragana microphylla* Lam. adapted to arid environment by increasing C and N contents of leaves (Xu et al., 2021; Li et al., 2023b). *Nitraria sibirica* promoted the accumulation of main osmotic adjustment substances (soluble sugar and proline) for osmotic regulation to adapt to arid saline-alkali environment (Li et al., 2019). Finally, based on transcriptome technology, some studies have shown that *NtCBL1* gene (*Nicotiana tabacum* Calcineurin B-like protein 1) may be involved in the response of *N. tangutorum* to salt and drought stress, while *NtCBL2* gene (*Nicotiana tabacum* Calcineurin B-like protein 2) plays a role under cold stress (Li et al., 2021). The genome-wide identification and bioinformatics analysis of the glutathione peroxidases (GPX) family of *N. sibirica* under salt stress showed that the expression of GPX gene was enhanced, the activity of glutathione peroxidase was increased, and the excessive ROS was eliminated, thereby enhancing the salt tolerance of plants (Lian et al., 2023).

There are few studies on the genetic adaptation mechanism of *N. sphaerocarpa* populations to heterogeneous environment. In this study, LFMM and RDA analysis showed that 3 out of 22 adaptive SNP loci were successfully aligned with functional genes related to plant biological processes and metabolic pathways (**Table 2**). Among them, protein phosphatase 2C (PP2C) have obvious regulation on abiotic stresses such as drought (Bhaskara et al., 2017), cold stress (Lenka et al., 2018) and salt stress (Manabe et al., 2008). In *Arabidopsis*, AtPP2CG1 was found to regulate salt stress in an ABA-dependent manner (Liu et al., 2012). In addition, studies have shown that PP2C can respond to drought and salt stress in crops (Li et al., 2013b; He et al., 2019; Yu et al., 2020). Therefore, PP2C members can indirectly regulate the growth and development of plants. Based on the candidate genes identified in this study, preliminary evidence suggests that physiological adaptation may be a main mechanism by which *N. sphaerocarpa* responds to environmental stress. However, due to the limited functional annotation of the candidate loci, further studies are required to validate the functions of these genes in order to elucidate the primary mechanisms underlying local adaptation in *N. sphaerocarpa.* This study analyzed the mechanism of *N. sphaerocarpa* adaptation to environment, aiming to establish a theoretical framework for understanding the molecular genetic processes involved in the plant’s local adaptive evolution driven by environmental factors.

## 5 Conclusions

In summary, this study focused on population genetic structure and local adaptation of *N. sphaerocarpa*, a superxerophytic constructive species in arid northwestern China. Using population genomics and landscape genomics analysis methods, the research aimed to elucidate the regulatory mechanism in the adaptive evolution of *N. sphaerocarpa* populations. The analysis revealed that an increasing genetic diversity of populations was shown from west to east. The 20 populations gathered into four lineages that originated during the Pleistocene period. Both geographical and environmental distances significantly impacted genetic distance, suggesting that long geographical distances played a key role in the genetic differentiation and fragmented distribution of *N. sphaerocarpa* populations. The primary environmental adaptation pressures faced by *N. sphaerocarpa* was from fluctuations in temperature and precipitation conditions. Physiological mechanism likely serving as the important mechanism for adapting to environmental stress.

## Data Availability

The datasets presented in this study can be found in online repositories. The names of the repository/repositories and accession number(s) can be found below: https://ngdc.cncb.ac.cn/gsa, CRA017020.
