# Supplementary material for: Genetic structure and local adaptation of Nitraria sphaerocarpa populations from arid northwestern China
Source: Front Plant Sci. 2025 Sep 3;16:1623235. doi: 10.3389/fpls.2025.1623235 (PMC12440915; doi:10.3389/fpls.2025.1623235)
Supplement: Supplementary file 1 [file DataSheet1.docx]

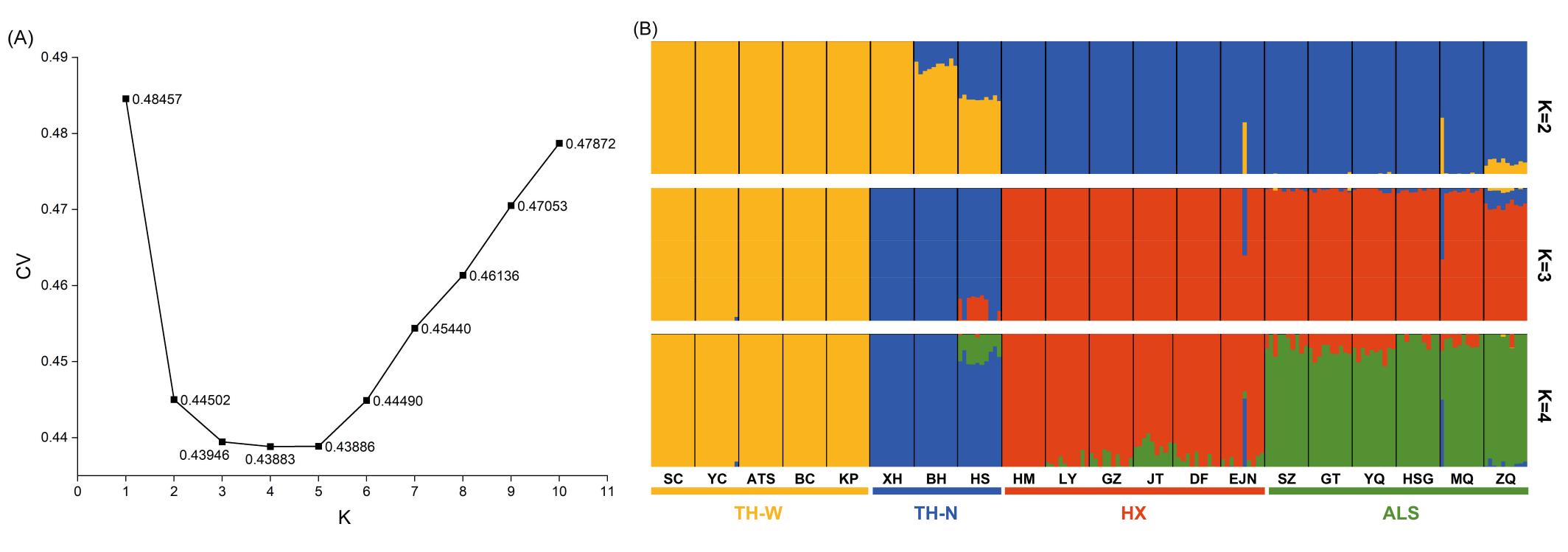


**Figure S1****.** **(A)** The Cross-Validation (CV) error distribution according to the number of clusters (K) by ADMIXTURE. **(B)** Admixture plot for all *N. sphaerocarpa* samples for K = 2–4. Each vertical bar represents an individual, the x-axis represents each population and four clusters and the y-axis represents the proportion of ancestors contained in each individual.


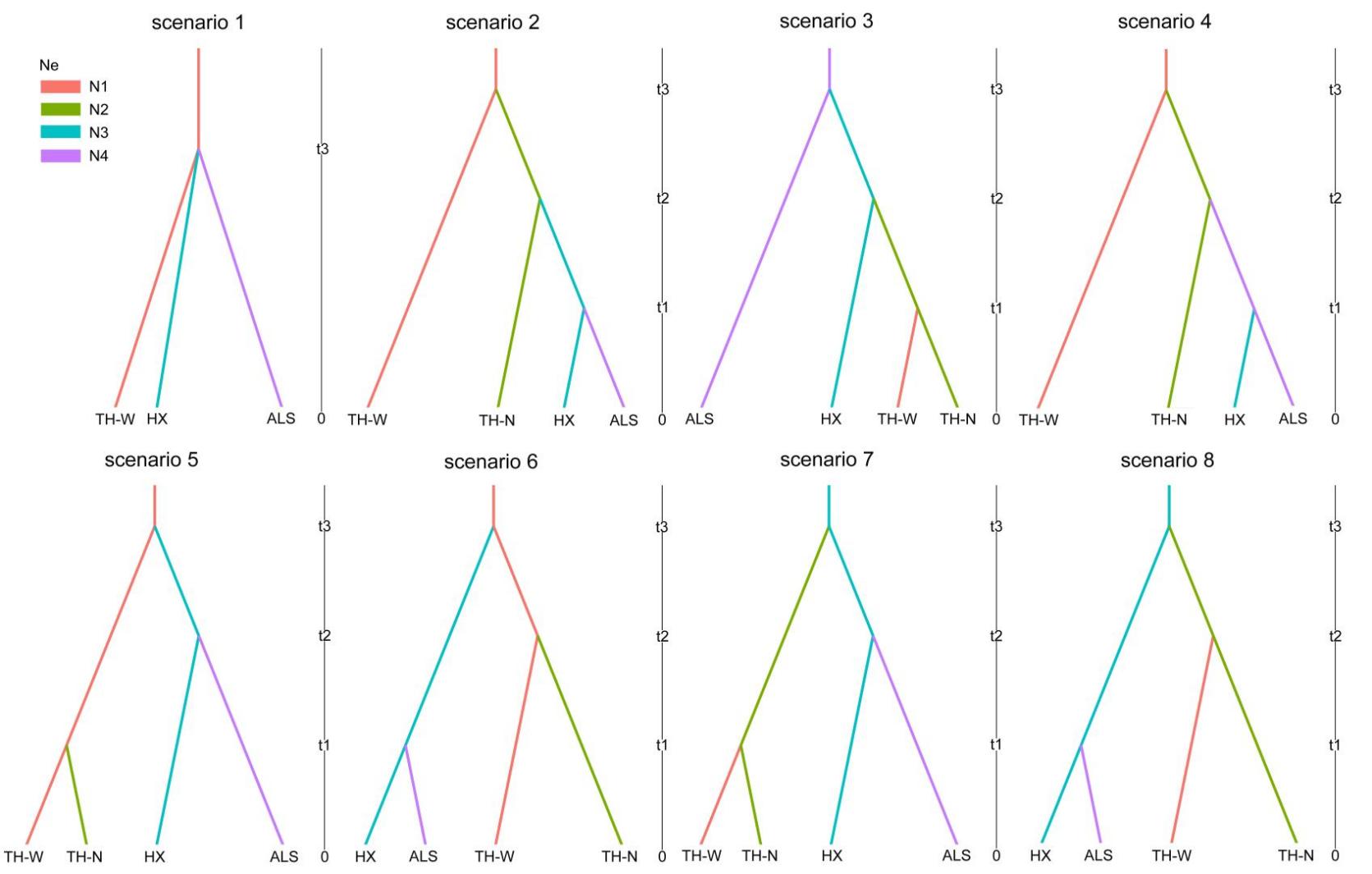


**Figure S2.** The 8 scenarios of population history of four lineages in *N. sphaerocarpa* with *DIYABC*. Each branch of different colors represents a lineage. N1, N2, N3 and N4 represent the effective population size of the four lineages. T1, t2 and t3 divergence times for the depicted event.


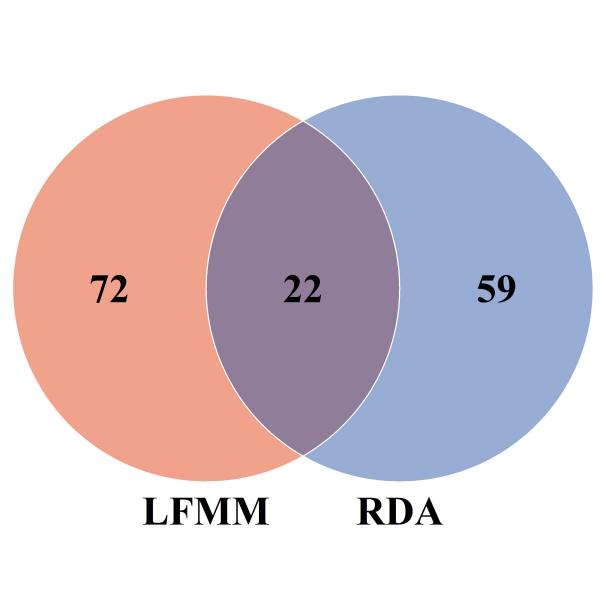


**Figure S3.** Venn diagram. It shows overlap of SNPs identified by latent factor mixed modeling (LFMM) and redundancy analysis (RDA).
