# Supplementary material for: Genetic structure and local adaptation of Nitraria sphaerocarpa populations from arid northwestern China
Source: Front Plant Sci. 2025 Sep 3;16:1623235. doi: 10.3389/fpls.2025.1623235 (PMC12440915; doi:10.3389/fpls.2025.1623235)
Supplement: Supplementary file 2 [file Table1.docx]

**Table S1** Details of sample locations, sample size and elevation for 20 *N. sphaerocarpa* populations.

| Region | Population | Number | Voucher No. | Location | n | Elevation(m) |
| --- | --- | --- | --- | --- | --- | --- |
| Tarim Basin | pgSC | 1 | pgbc-SC-0001 | Yarkant, Xinjiang | 10 | 1295 |
|  | pgATS | 2 | pgbc-ATS-0001 | Artux, Xinjiang | 10 | 1721 |
|  | pgYC | 3 | pgbc-YC-0001 | Qaghiliq, Xinjiang | 10 | 1570 |
|  | pgBC | 4 | pgbc-BC-0001 | Marabishi, Xinjiang | 10 | 1073 |
|  | pgKP | 5 | pgbc-KP-0001 | Kalpin, Xinjiang | 10 | 1076 |
|  | pgXH | 6 | pgbc-XH-0001 | Toksu, Xinjiang | 10 | 919 |
|  | pgBH | 7 | pgbc-BH-0001 | Bagrax, Xinjiang | 10 | 1045 |
|  | pgHS | 8 | pgbc-HS-0001 | Hoxud, Xinjiang | 10 | 1115 |
| Hami Basin | pgHM | 9 | pgbc-HM-0001 | Hami, Xinjiang | 10 | 1384 |
| Hexi Corridor | pgLY | 10 | pgbc-LY-0001 | Liuyuan, Gansu | 10 | 1733 |
|  | pgGZ | 11 | pgbc-GZ-0001 | Guazhou, Gansu | 10 | 1114 |
|  | pgJT | 12 | pgbc-JT-0001 | Jinta, Gansu | 10 | 1034 |
|  | pgSZ | 13 | pgbc-SZ-0001 | Suzhou, Gansu | 10 | 1406 |
|  | pgGT | 14 | pgbc-GT-0001 | Gaotai, Gansu | 10 | 1395 |
|  | pgHSG | 15 | pgbc-HSG-0001 | Hongshagang, Gansu | 10 | 1321 |
|  | pgMQ | 16 | pgbc-MQ-0001 | Minqin, Gansu | 10 | 1362 |
| Alxa Desert | pgDF | 17 | pgbc-DF-0001 | Dongfeng Town, Inner Mongolia | 10 | 953 |
|  | pgEJN | 18 | pgbc-EJN-0001 | Ejin Banner, Inner Mongolia | 10 | 913 |
|  | pgYQ | 19 | pgbc-YQ-0001 | Alxa Right Banner, Inner Mongolia | 10 | 1530 |
|  | pgZQ | 20 | pgbc-ZQ-0001 | Alxa Left Banner, Inner Mongolia | 10 | 1087 |

**Table S2** Pairwise genetic differentiation (*F_ST_*) of 20 *N. sphaerocarpa* populations.

| Sample | pgATS | pgBC | pgBH | pgDF | pgEJN | pgGT | pgGZ | pgHM | pgHS | pgHSG | pgJT | pgKP | pgLY | pgMQ | pgSC | pgSZ | pgXH | pgYC | pgYQ | pgZQ |
| --- | --- | --- | --- | --- | --- | --- | --- | --- | --- | --- | --- | --- | --- | --- | --- | --- | --- | --- | --- | --- |
| pgATS |  |  |  |  |  |  |  |  |  |  |  |  |  |  |  |  |  |  |  |  |
| pgBC | 0.19 |  |  |  |  |  |  |  |  |  |  |  |  |  |  |  |  |  |  |  |
| pgBH | 0.46 | 0.47 |  |  |  |  |  |  |  |  |  |  |  |  |  |  |  |  |  |  |
| pgDF | 0.40 | 0.39 | 0.41 |  |  |  |  |  |  |  |  |  |  |  |  |  |  |  |  |  |
| pgEJN | 0.39 | 0.37 | 0.40 | 0.04 |  |  |  |  |  |  |  |  |  |  |  |  |  |  |  |  |
| pgGT | 0.38 | 0.38 | 0.40 | 0.16 | 0.14 |  |  |  |  |  |  |  |  |  |  |  |  |  |  |  |
| pgGZ | 0.38 | 0.37 | 0.40 | 0.07 | 0.08 | 0.12 |  |  |  |  |  |  |  |  |  |  |  |  |  |  |
| pgHM | 0.39 | 0.38 | 0.40 | 0.07 | 0.07 | 0.15 | 0.04 |  |  |  |  |  |  |  |  |  |  |  |  |  |
| pgHS | 0.43 | 0.43 | 0.26 | 0.36 | 0.34 | 0.34 | 0.34 | 0.36 |  |  |  |  |  |  |  |  |  |  |  |  |
| pgHSG | 0.38 | 0.37 | 0.39 | 0.15 | 0.14 | 0.08 | 0.12 | 0.14 | 0.33 |  |  |  |  |  |  |  |  |  |  |  |
| pgJT | 0.37 | 0.37 | 0.40 | 0.03 | 0.06 | 0.12 | 0.07 | 0.08 | 0.34 | 0.11 |  |  |  |  |  |  |  |  |  |  |
| pgKP | 0.20 | 0.12 | 0.46 | 0.39 | 0.37 | 0.38 | 0.36 | 0.38 | 0.45 | 0.37 | 0.37 |  |  |  |  |  |  |  |  |  |
| pgLY | 0.37 | 0.37 | 0.38 | 0.06 | 0.05 | 0.13 | 0.00 | 0.03 | 0.33 | 0.11 | 0.06 | 0.37 |  |  |  |  |  |  |  |  |
| pgMQ | 0.35 | 0.35 | 0.38 | 0.12 | 0.13 | 0.08 | 0.10 | 0.14 | 0.31 | 0.04 | 0.10 | 0.35 | 0.11 |  |  |  |  |  |  |  |
| pgSC | 0.25 | 0.26 | 0.47 | 0.41 | 0.39 | 0.39 | 0.37 | 0.39 | 0.46 | 0.39 | 0.39 | 0.26 | 0.37 | 0.37 |  |  |  |  |  |  |
| pgSZ | 0.39 | 0.38 | 0.41 | 0.17 | 0.16 | 0.08 | 0.13 | 0.15 | 0.34 | 0.11 | 0.15 | 0.38 | 0.13 | 0.10 | 0.40 |  |  |  |  |  |
| pgXH | 0.53 | 0.50 | 0.59 | 0.49 | 0.44 | 0.48 | 0.46 | 0.47 | 0.58 | 0.45 | 0.47 | 0.49 | 0.47 | 0.44 | 0.51 | 0.48 |  |  |  |  |
| pgYC | 0.27 | 0.29 | 0.47 | 0.41 | 0.39 | 0.40 | 0.37 | 0.39 | 0.46 | 0.39 | 0.38 | 0.29 | 0.38 | 0.36 | 0.23 | 0.39 | 0.50 |  |  |  |
| pgYQ | 0.38 | 0.37 | 0.40 | 0.12 | 0.12 | 0.07 | 0.10 | 0.14 | 0.34 | 0.06 | 0.09 | 0.37 | 0.11 | 0.06 | 0.39 | 0.10 | 0.46 | 0.39 |  |  |
| pgZQ | 0.41 | 0.40 | 0.44 | 0.24 | 0.24 | 0.24 | 0.26 | 0.24 | 0.40 | 0.21 | 0.24 | 0.40 | 0.23 | 0.20 | 0.41 | 0.25 | 0.51 | 0.42 | 0.23 |  |

**Table S3** The corresponding 13 bioclimatic data of each population after removing collinearity.

| Sample | **bio1**  **(℃×10)** | **bio2**  **(℃)** | bio3  (%) | **bio4**  **（*SD*×100）** | bio8  (℃**×**10) | bio9  (℃**×**10) | bio10  (℃**×**10) | **bio11**  **(℃×10)** | bio14 (mm) | **bio15 (%)** | bio17 (mm) | **bio18 (mm)** | bio19  (mm) |
| --- | --- | --- | --- | --- | --- | --- | --- | --- | --- | --- | --- | --- | --- |
| pgATS | **76** | **110** | 27 | **9684** | 196 | -72 | 196 | **-72** | 1 | **78** | 4 | **58** | 4 |
| pgBC | **125** | **114** | 26 | **10866** | 254 | 70 | 254 | **-45** | 0 | **94** | 2 | **39** | 3 |
| pgBH | **98** | **119** | 26 | **11299** | 239 | -36 | 239 | **-72** | 1 | **92** | 3 | **51** | 5 |
| pgDF | **98** | **127** | 25 | **12994** | 261 | -64 | 269 | **-92** | 0 | **107** | 0 | **30** | 0 |
| pgEJN | **98** | **126** | 24 | **13143** | 262 | -66 | 270 | **-94** | 0 | **107** | 0 | **28** | 0 |
| pgGT | **86** | **118** | 28 | **10009** | 213 | -59 | 218 | **-59** | 1 | **99** | 3 | **87** | 3 |
| pgGZ | **108** | **124** | 27 | **11321** | 255 | -32 | 255 | **-58** | 1 | **90** | 3 | **32** | 3 |
| pgHM | **77** | **123** | 26 | **12043** | 233 | -65 | 233 | **-100** | 1 | **88** | 3 | **33** | 3 |
| pgHS | **84** | **115** | 26 | **10840** | 220 | -41 | 220 | **-77** | 1 | **97** | 3 | **58** | 5 |
| pgHSG | **95** | **121** | 26 | **11294** | 236 | -34 | 243 | **-68** | 0 | **98** | 2 | **58** | 2 |
| pgJT | **99** | **128** | 25 | **12546** | 256 | -46 | 263 | **-85** | 0 | **104** | 1 | **37** | 2 |
| pgKP | **116** | **111** | 26 | **10851** | 245 | 61 | 245 | **-52** | 0 | **95** | 2 | **40** | 3 |
| pgLY | **65** | **124** | 26 | **11549** | 215 | -78 | 215 | **-105** | 1 | **94** | 3 | **28** | 3 |
| pgMQ | **94** | **122** | 27 | **11113** | 232 | -33 | 239 | **-68** | 0 | **101** | 2 | **68** | 2 |
| pgSC | **116** | **105** | 26 | **9818** | 212 | -35 | 235 | **-35** | 1 | **79** | 3 | **45** | 3 |
| pgSZ | **82** | **119** | 28 | **10101** | 209 | -65 | 214 | **-65** | 1 | **105** | 3 | **75** | 3 |
| pgXH | **115** | **106** | 24 | **11419** | 251 | -21 | 251 | **-60** | 1 | **67** | 3 | **33** | 5 |
| pgYC | **99** | **107** | 29 | **8973** | 152 | -3 | 210 | **-37** | 1 | **77** | 3 | **35** | 3 |
| pgYQ | **81** | **121** | 26 | **11472** | 225 | -64 | 231 | **-85** | 1 | **101** | 3 | **74** | 3 |
| pgZQ | **97** | **124** | 26 | **11959** | 245 | -55 | 253 | **-78** | 1 | **106** | 3 | **80** | 3 |

Note: Bio 1, annual mean temperature; bio 2, mean diurnal range; bio 3, isothermality; bio 4, temperature seasonality; bio 8, mean temperature of wettest quarter; bio 9, mean temperature of driest quarter; bio 10, mean temperature of warmest quarter; bio 11, mean temperature of coldest quarter;bio 14, precipitation of driest month; bio 15, precipitation seasonality; bio 17, precipitation of driest quarter; bio 18, precipitation of warmest quarter; bio 19, precipitation of coldest quarter; 13 bioclimatic variables used in GF analysis. In addition, font coarsening represents the top 6 environmental factors in GF ranking, which are used for LFMM and RDA analysis.
